# Concurrent scale-free and small-world networks support criticality in cortical ensembles

**DOI:** 10.1186/1471-2202-14-S1-P264

**Published:** 2013-07-08

**Authors:** Paolo Massobrio, Valentina Pasquale, Sergio Martinoia

**Affiliations:** 1 Department of Informatics, Bioengineering, Robotics and Systems Engineering (DIBRIS), University of Genova, Genova, 16145, Italy; 2 Department of Neuroscience and Brain Technologies - NTECH, Istituto Italiano di Tecnologia (IIT), Via Morego 30, 16163, Genova, Italy

## 

Nowadays, it is widely accepted that structural features of cortical networks are tightly linked to aspects of brain function, playing a crucial role in determining which electrophysiological patterns (and thus, brain states) can and cannot occur. In this work, we investigated the interplay between network topology and spontaneous dynamics within the framework of neuronal avalanches and self-organized criticality [1].

The aim of this study is to sustain the hypothesis that the emergence of critical states, which in their turn would optimize functional properties in the cortex, is supported by specific complex network topologies.

We followed a computational approach by developing large-scale network models mimicking the electrophysiological patterns (i.e., spiking and bursting activity) displayed by *in vitro *cortical networks coupled to Micro-Electrode Arrays (MEAs). In this preparation, neurons are able to freely re-create networks that exhibit complex and highly variable spatio-temporal patterns of activity [2]. As neurons grow without external constraints and self-organize depending on many parameters, we do not have any clue about the resulting morphological connectivity. Moreover, some cultures actually exhibit scale-free distributions of neuronal avalanches, a hallmark of SOC, thus demonstrating that they preserve self-organization properties featured by *in vivo*-formed cell assemblies [3]. Therefore, cortical networks actually show different states (critical, subcritical or supercritical), but their relationship with the underlying connectivity remains unknown [4]. Due to the difficulties of determining the network topology of our cultures from a limited number of recording sites (60 microelectrodes), we took advantage of a computational model consisting of a neuronal network made up of 1024 Izhikevich neurons [5]. Network topologies were designed following the canonical architectures scale-free, random, and small-world [6]. We simulated the spontaneous activity of such neuronal networks, by sweeping the most common parameters used to characterize these graphs, such as clustering coefficient, connection density, etc. [7]. The main finding which emerges is that although all the network configurations determine a mix of spiking, and bursting activity, the scale-free with small-world features display a critical behavior.
